# Case report: Primary pulmonary rhabdomyosarcoma exhibiting epithelial morphology and unusual immunophenotype – A significant diagnostic pitfall

**DOI:** 10.1016/j.rmcr.2026.102389

**Published:** 2026-02-18

**Authors:** Zonghua Wen, Wenting Li, Yao Liu, Hongyun Chen, Xuxu Wen, Jun Wang, Yungang Lv, Ruodai Wu, Yuhong Meng

**Affiliations:** a Department of Pathology, Shenzhen University General Hospital, Xueyuan AVE 1298, Nanshan District, Shenzhen, Guangdong Province, 518055, China; b Department of Thoracic Surgery, Shenzhen University General Hospital. Xueyuan AVE 1298, Nanshan District, Shenzhen, Guangdong Province, 518055, China; c Department of Radiology, Shenzhen University General Hospital, Xueyuan AVE 1298, Nanshan District, Shenzhen, Guangdong Province, 518055, China

**Keywords:** Pulmonary, Rhabdomyosarcoma, Epithelial, TTF-1

## Abstract

Primary pulmonary rhabdomyosarcoma(PPRMS) is an extremely rare neoplasm.Herin,we report a case of a 60-year-old male who presented with a one-week history of progressive chest tightness and non-radiating chest pain. Contrast-enhanced thoracic computed tomography (CT) demonstrated a large,11cm mass in the right inferior lung lobe. Intraoperative frozen-section analysis initially misclassified the lesion as a poorly differentiated carcinoma. The patient subsequently underwent thoracoscopic right lower lobectomy with systematic mediastinal lymph node dissection. Histological examination demonstrated sheets of uniformly sized epithelioid cells arranged in an organoid nesting growth pattern, with areas of geographic necrosis and no definitive morphological features indicative of rhabdomyoblastic differentiation. Immunohistochemical(IHC) staining confirmed diffuse positivity for skeletal muscle lineage markers(desmin, myogenin, MyoD1) in the tumor cells, thereby establishing a definitive diagnosis of rhabdomyosarcoma. Notably, this case showed diffuse expression of thyroid transcription factor 1 (TTF-1) using both the 8G7G3 and SPT24 clones, alongside immunoreactivity for neuroendocrine markers(CD56, PGP9.5) and focal expression of epithelial markers(AE1/AE3, CAM5.2), This immunophenotypic profile may mimic that of poorly differentiated neuroendocrine carcinoma, contributing to diagnostic confusion. The constellation of these unusual histological and immunophenotypic features presents a substantial diagnostic challenge. We herein elaborate on these findings to enhance clinical awareness and facilitate accurate diagnosis in future cases. To the best of our knowledge, To the best of our knowledge, this is the first reported case of epithelioid rhabdomyosarcoma demonstrating diffuse and strong nuclear TTF-1 immunoreactivity across two distinct antibody clones.

## 1 Introduction

According to the current World Health Organization (WHO) classification, rhabdomyosarcoma (RMS) is categorized into four main subtypes: embryonal RMS (ERMS), alveolar RMS (ARMS), pleomorphic RMS (PRMS) and spindle cell/sclerosing RMS [1]. In 1989, Seidal et al. [2] described a case of RMS that, while lacking classic microscopic features of rhabdomyoblastic differentiation, demonstrated ultrastructural and immunohistochemical evidence of myogenic lineage commitment. Given its distinct epithelioid morphology, they coined the term “epithelioid rhabdomyosarcoma” (eRMS) to describe this variant. Since this initial report, a growing number of similar cases have been documented in the literature [[3], [4], [5], [6], [7], [8]]. Even among this rare subset, primary pulmonary epithelioid represents an exceptionally rare pathological entity. Herein, we describe a case of primary pulmonary eRMS that demonstrated diffuse, intense nuclear immunoreactivity for thyroid transcription factor-1(TTF-1). Notably, the tumor also showed immuno reactivity for a subset of neuroendocrine markers, as well as focal expression of epithelial markers. This report details our findings, emphasizing the unique immunophenotypic profile of this case and the diagnostic challenges it poses, to enhance clinical awareness of this rare entity.

## 2 Case presentation

### 2.1 Clinical presentation

**Fig. 1:**
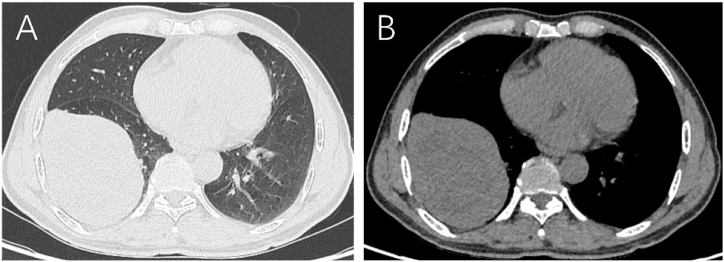
Computed tomography (CT) revealed an 11-cm mass in the right lower lobe of the lung to have heterogeneous density, relatively clear margins, and no calcification. A (Lung Window), B (Mediastinal Window).

A 60-year-old male presented with a one-week history of progressive chest tightness and non-radiating chest pain. His past medical history was unremarkable, with no prior diagnosis of malignancy. Contrast-enhanced thoracic computed tomography (CT) demonstrated an 11cm mass in the right lower lobe of the lung (Fig. 1). No evidence of additional primary lesions or metastatic disease was identified on staging imaging. Intraoperative frozen section analysis initially misclassified the lesion as poorly differentiated carcinoma. The patient subsequently underwent a thoracoscopic resection of the right inferior lung lobe combined with systematic mediastinal lymph node dissection.

### 2.2 Pathological features

**Fig. 2:**
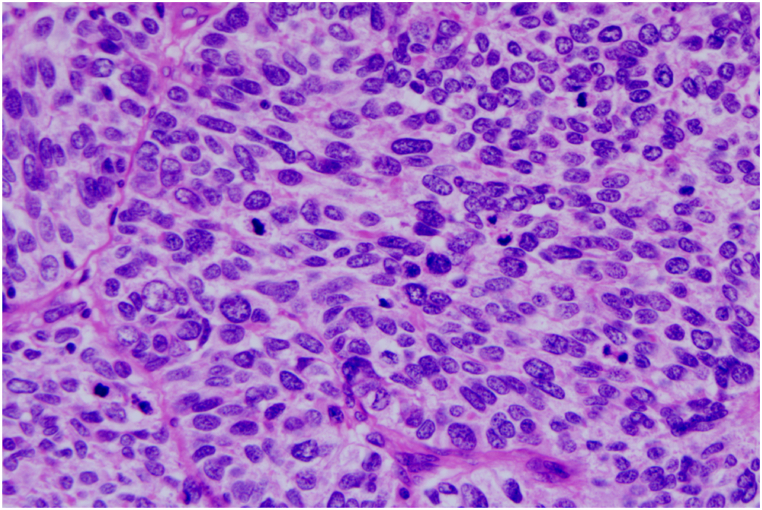
Histological features of epithelioid RMS. (H&E, original magnification × 400). This image demonstrates sheets of uniformly-sized epithelioid cells arranged in an organoid-nesting trabecular growth pattern, with areas of multifocal geographic necrosis visible. At higher magnification, the lesion consists of large polygonal cells with small nucleoli, a moderate amount of cytoplasm, and no obvious morphological features of rhabdomyoblastic differentiation; the chromatin appears coarsely granular or stippled, with some cells showing a vesicular texture.

Gross pathological examination revealed a poorly circumscribed tumor with a maximum diameter of 11cm, a hemorrhagic cut surface, and focal areas of necrosis. Histological examination demonstrated tumor cells arranged in an organoid nesting trabecular growth pattern, accompanied by multifocal geographic necrosis. The lesion was composed of sheets and nests of large polygonal cells with small nucleoli and a moderate amount of cytoplasm, lacking definitive morphological features indicative of rhabdomyoblastic differentiation, and exhibited high mitotic activity(60–80 mitoses per 10 high-power fields [HPF], 40 × objective). The chromatin appeared coarsely granular or stippled, with some cells showing a vesicular texture and prominent small nucleoli nucleoli (Fig. 2).

### 2.3 Immunohistochemical and molecular findings

**Fig. 3:**
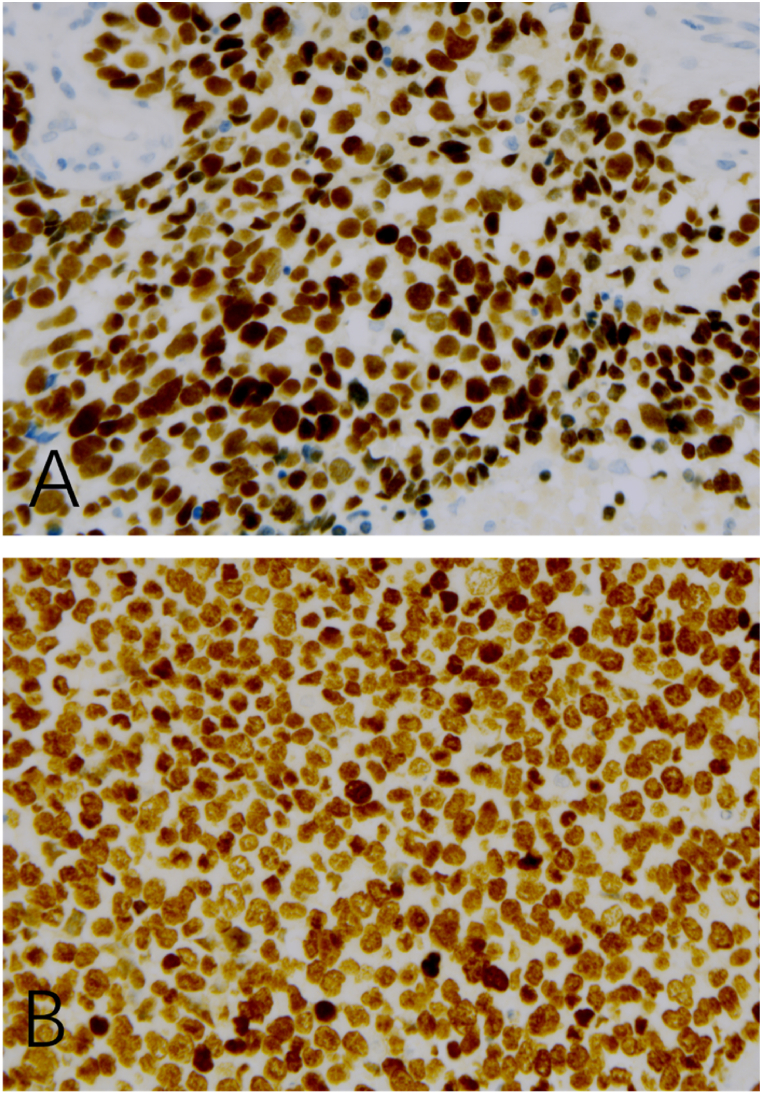
Immunophenotypes of epithelioid RMS, demonstrating TTF-1 expression with two distinct antibody clones (IHC, original magnification × 400). (A) Diffuse and strong nuclear positivity for TTF-1 is observed with the 8G7G3/1 antibody clone. (B) Diffuse and strong nuclear positivity for TTF-1 is similarly demonstrated with the SPT24 antibody clone, confirming consistent immunoreactivity across both clones.

A complete immunohistochemical (IHC) panel was performed, with details of antibody clones, staining extent, and intensity summarized in Table 1.Immunohistochemical analysis revealed diffuse strong positivity for desmin, myogenin, MyoD1, TTF-1 with the 8G7G3/1 antibody clone (Fig. 3A), TTF-1 with the SPT24 antibody clone (Fig. 3B),CD56, PGP9.5, p53, bcl-2 and BRG1(SMARCA4), INI-1. Focal staining was observed for pancytokeratin (AE1/AE3), and cytokeratin 8/18 (CAM5.2). The Ki-67 proliferation index was determined to be 80–90%. Tumor cells were negative for INSM1, SSTR2, synaptophysin, chromogranin, neuron-specific enolase (NSE), NapsinA, p40, p63, NUT, s100, Erg, FLI-1, SALL-4, CK7, CK5/6, and CD34. Fluorescence in situ hybridization (FISH) was negative for FOXO1 gene rearrangement.

**Table 1:** Antibodies used.

Antibodies	Clone	Staining Extent	Staining Intensity
desmin	DE-R-11	Diffuse (>90% of cells)	Strong (3+)
myogenin	MX078	Diffuse (>90% of cells)	Strong (3+)
MyoD1	EP212	Diffuse (>90% of cells)	Strong (3+)
TTF-1	8G7G3	Diffuse (>90% of cells)	Strong (3+)
TTF-1	SPT24	Diffuse (>90% of cells)	Strong (3+)
pancytokeratin	AE1-AE3	Focal (2-3% of cells)	Weak (1+)
cytokeratin 8/18	OT18D4/UMAB50	Focal (2-3% of cells)	Weak (1+)
INSM1	MRQ-70	Negative (<1% of cells)	Negative (0)
SSTR2	EP149	Negative (<1% of cells)	Negative (0)
synaptophysin	EP158	Negative (<1% of cells)	Negative (0)
chromogranin	OTI1G3	Negative (<1% of cells)	Negative (0)
neuron-specific enolase	OTI10D1	Negative (<1% of cells)	Negative (0)
NapsinA	OT185	Negative (<1% of cells)	Negative (0)
p40	OTI12A10	Negative (<1% of cells)	Negative (0)
NUT	OTIR6H1	Negative (<1% of cells)	Negative (0)
s100	15E2E2+4C4.9	Negative (<1% of cells)	Negative (0)
FLI-1	G146-22	Negative (<1% of cells)	Negative (0)
SALL-4	6E3	Negative (<1% of cells)	Negative (0)
Ki-67	MIB1	Diffuse (80∼90% of cells)	Strong (3+)
BRG1(SMARCA4)	E8V5B	Diffuse (>90% of cells)	Strong (3+)
INI-1	OTIR4G9	Diffuse (>90% of cells)	Strong (3+)

### 2.4 Diagnosis and differential diagnosis

Notably, this case cannot be categorized into any of the four World Health Organization (WHO)-recognized rhabdomyosarcoma subtypes (embryonal, alveolar, pleomorphic, spindle cell/sclerosing). It lacks the myxoid stroma and rhabdomyoblastic differentiation of embryonal RMS; shows no alveolar architecture or PAX3/PAX7-FOXO1 gene rearrangements typical of alveolar RMS; does not exhibit the marked cellular pleomorphism of pleomorphic RMS; and lacks the spindle cell morphology or sclerotic stroma of spindle cell/sclerosing RMS.

This case demonstrates strong clinicopathological concordance with the epithelioid rhabdomyosarcoma (ERMS) phenotype detailed in the reference study. Histologically, the lesion consists of a diffuse sheet-like proliferation of uniform epithelioid cells, characterized by large vesicular nuclei, prominent nucleoli, abundant amphophilic-to-eosinophilic cytoplasm, and high mitotic activity—findings consistent with ERMS morphology. Immunohistochemical studies confirm definitive skeletal muscle differentiation, establishing the diagnosis of rhabdomyosarcoma.

The primary differential diagnoses for epithelioid rhabdomyosarcoma (ERMS) include multiple epithelioid-morphology malignancies, particularly poorly differentiated carcinomas (including neuroendocrine carcinomas). Despite its epithelioid morphology, the present tumor exhibits mesenchymal features—poorly defined cell borders, cohesive cell distribution, and delicate fibrovascular septa separating cell nests. Immunohistochemically, focal pancytokeratin expression contrasts sharply with diffuse, strong positivity for myogenic markers (desmin, myogenin, MyoD1), enabling definitive distinction from poorly differentiated carcinomas.

Large-cell neuroendocrine carcinoma (LCNEC) represents a key differential consideration, given overlapping morphological features, despite fundamental differences in histogenesis and immunoprofiles. LCNEC typically presents with nested, trabecular, or organoid architecture, well-demarcated cell borders, abundant eosinophilic/amphophilic cytoplasm, pleomorphic nuclei with prominent nucleoli, high mitotic activity, and geographic necrosis. However, it lacks the mesenchymal hallmarks observed herein (cohesive clusters with ill-defined borders, fusion-like distribution, and delicate fibrovascular septa), instead showing distinct epithelial architecture with intercellular junctions. Immunohistochemically, LCNEC is characterized by diffuse, strong expression of neuroendocrine markers (synaptophysin, chromogranin A, CD56), diffuse pancytokeratin positivity. In contrast, the present case shows only focal pancytokeratin staining, absent neuroendocrine marker expression, and diffuse myogenic marker positivity—findings consistently negative in LCNEC.

Intraoperative frozen-section misdiagnosis of this ERMS case constitutes a critical teaching point, highlighting challenges in differentiating rare mesenchymal tumors from common epithelial neoplasms (e.g., LCNEC) intraoperatively. Morphological overlap (epithelioid features, nested growth, geographic necrosis) with LCNEC and high-grade poorly differentiated carcinomas, combined with frozen-section limitations (suboptimal fixation, tissue artifacts, limited sampling), obscured subtle mesenchymal features evident in paraffin sections. The unavailability of intraoperative immunohistochemistry—essential for distinguishing myogenic vs. epithelial/neuroendocrine markers—further impeded accurate classification, as did limited familiarity with the rare frozen-section phenotype of pulmonary ERMS, which biased interpretation toward more prevalent epithelial tumors. This case underscores that frozen-section analysis prioritizes urgent intraoperative queries (e.g., margin status) over definitive subtyping of rare entities, and emphasizes the necessity of correlating frozen findings with post-operative comprehensive paraffin-section immunohistochemistry and molecular testing to confirm the diagnosis.

### 2.5 Treatment and follow-up

During the follow-up period, the patient was regularly monitored for disease progression. The patient had previously undergone thoracoscopic right lower lobectomy combined with mediastinal lymph node dissection. However, due to family financial constraints, the patient did not receive any postoperative adjuvant therapy (including chemotherapy, targeted therapy, or immunotherapy) as recommended by the multidisciplinary team. Unfortunately, the patient succumbed to the disease six months postoperatively, and the exact cause of death remained undetermined due to the lack of a detailed autopsy and limited follow-up medical records.

## 3 Discussion

Epithelioid rhabdomyosarcoma (RMS), a rare and aggressive variant of RMS, is often misdiagnosed as poorly differentiated neuroendocrine carcinoma by the referring pathologist due to its epithelioid morphology, aberrant immunophenotype [9]. In the majority of cases, no distinct morphological characteristics indicative of rhabdomyoblastic differentiation are evident. The characteristic traits of rhabdomyosarcoma (RMS), such as the presence of rhabdomyoblasts in straps or spiders or the typical alveolar structure with giant wreath-shaped cells, are notably absent in epithelioid RMS. As a result, relying solely on histological examination, it is extremely challenging to identify the tumour as RMS. The rhabdomyoblastic differentiation is ultimately verified through immunoreactivity for desmin and myogenin. The utilization of myogenic markers, namely desmin, myogenin, and MyoD1, proves beneficial in differentiating epithelioid rhabdomyosarcoma (RMS) from poorly differentiated carcinomas.

Previous reports have documented neuroendocrine carcinomas with skeletal muscle differentiation, typically showing biphasic differentiation with an intimate intermingling of neuroendocrine carcinoma and spindle cell sarcoma components [[10], [11], [12], [13]]. Guillermo et al. [10] reported that muscle markers can be expressed in neuroendocrine carcinoma, with positive levels of desmin, myogenin, and MyoD1 observed in 35%, 35%, and 48%, respectively. However, staining is usually focal and affects less than 10% of tumor cells. In contrast, our case showed diffuse positivity for desmin, myogenin, and MyoD1, while more specific neuroendocrine markers were negative, which supports the diagnosis of rhabdomyosarcoma.

Aberrant expression of epithelial and neuroendocrine markers in rhabdomyosarcoma (RMS) is not infrequent [14]. Bahrami et al. [15] reported a large cohort of cases of rhabdomyosarcoma exhibiting coexpression of these markers. Specifically, 50% of the cases showed positivity for wide-spectrum cytokeratin, while 52% were positive for Cam5.2. Moreover, CD56 expression was detected in 97% of the cases. Notably, 43% of the cases expressed at least one specific neuroendocrine marker: 32% were positive for synaptophysin, 22% for chromogranin A, and 11% for both. These findings have been corroborated by other independent studies [9,16,17], highlighting the importance of these immunophenotypic characteristics in the characterization of RMS.

A comprehensive literature search was conducted across multiple databases, including PubMed, Web of Science, and Google Scholar, to identify all published cases documenting thyroid transcription factor-1 (TTF-1) expression in tumors. TTF-1 expression is predominantly observed in primary pulmonary adenocarcinomas, thyroid carcinomas, and neuroendocrine tumors [18], as well as in a small subset of carcinomas arising from other anatomical sites [[19], [20], [21]]. Rare instances of TTF-1 expression have been reported in central nervous system tumors [22], peripheral T-cell lymphomas [23], and diffuse large B-cell lymphomas [24]. Occasional TTF-1 expression has also been documented in mesenchymal-origin tumors (e.g., mesothelioma [25]) and embryonal tumors (e.g., nephroblastomas [26]). Notably, focal immunohistochemical expression of TTF-1 has been described in two previously reported cases of primary pulmonary rhabdomyosarcoma [27]. These two cases share striking similarities with our case regarding the primary tumor site, morphological characteristics, and core immunohistochemical profiles. The key discriminative feature lies in the expression pattern of TTF-1: the previously reported cases exhibited focal TTF-1 expression, whereas our case displays diffuse and strong TTF-1 expression.

Our case is the first to report diffuse and intense expression of TTF-1 in rhabdomyosarcoma, increasing the risk of misdiagnosis, especially as a high-grade neuroendocrine carcinoma.

## 4 Conclusion

In summary, we present a case of primary pulmonary rhabdomyosarcoma. This tumor exhibits epithelial morphology, shows aberrant expression of TTF-1, and has coexpression of epithelial and neuroendocrine markers. Such characteristics may lead to misdiagnosing the tumor as poorly differentiated neuroendocrine carcinoma. Familiarity with its clinicopathological characteristics and immunohistochemical profiles will help pathologists arrive at the correct diagnosis.

## CRediT authorship contribution statement

**Zonghua Wen:** Writing – original draft. **Wenting Li:** Formal analysis. **Yao Liu:** Data curation. **Hongyun Chen:** Methodology. **Xuxu Wen:** Methodology. **Jun Wang:** Resources. **Yungang Lv:** Methodology. **Ruodai Wu:** Methodology. **Yuhong Meng:** Writing – review & editing.

## Ethical approval and informed consent statements

The study was conducted in accordance with ethical principles and informed consent was obtained from the patient.

## Declaration of competing interest

The authors declare the following financial interests/personal relationships which may be considered as potential competing interests: Reports a relationship with that includes:. Has patent pending to. If there are other authors, they declare that they have no known competing financial interests or personal relationships that could have appeared to influence the work reported in this paper.
